# Use of traditional and complementary medicine among Norwegian cancer patients in the seventh survey of the Tromsø study

**DOI:** 10.1186/s12906-019-2762-7

**Published:** 2019-11-29

**Authors:** Agnete Egilsdatter Kristoffersen, Trine Stub, Ann Ragnhild Broderstad, Anne Helen Hansen

**Affiliations:** 1 National Research Center in Complementary and Alternative Medicine (NAFKAM), Department of Community Medicine, Faculty of Health Science, UiT The Arctic University of Norway, N-9037 Tromsø, Norway; 2 Centre for Sami Health Research, Department of Community Medicine, Faculty of Health Science, UiT The Arctic University of Norway, Tromsø, Norway; 3 Department of Medicine, University Hospital of North Norway, Harstad, Norway; 4 Centre for Quality Improvement and Development, University Hospital of North Norway, Tromsø, Norway; 5 Department of Community Medicine, Faculty of Health Sciences, UiT The Arctic University of Norway, Tromsø, Norway

**Keywords:** Cancer, Complementary and alternative medicine, CAM, Traditional and complementary medicine, T&CM, Complementary therapies, Traditional medicine, Traditional healing, Spiritual healing, Religious healing, Norway, The Tromsø study

## Abstract

**Background:**

Traditional and complementary medicine (T&CM) is commonly used by cancer patients in Northern Norway, in particular spiritual forms like traditional healing. T&CM is mainly used complementary to conventional cancer treatment and is rarely discussed with conventional health care providers, increasing the risk of negative interaction with conventional cancer care. The aim of this study was to investigate the use of T&CM among cancer patients in Tromsø, and to investigate the differences in T&CM use between people living with cancer, people with cancer previously, and people without a history of cancer.

**Method:**

Data was drawn from the seventh survey of the Tromsø study conducted in 2015–2016. All inhabitants of Tromsø aged 40 and above were invited to participate (*n* = 32,591) of whom *n* = 21,083 accepted the invitation (response rate 65%). Data was collected thorough three self-administered questionnaires and a comprehensive clinical examination. Pearson chi-square tests, Fisher exact tests and one-way ANOVA tests were used to describe differences between the groups while binary logistic regressions were used for adjusted values.

**Results:**

Eight percent of the participants (*n* = 1636) reported to have (*n* = 404) or have had (*n* = 1232) cancer. Of the participants with cancer at present 33.4% reported use of T&CM within the last year, 13.6% had consulted a T&CM provider, 17.9% had used herbal medicine/natural remedies and 6.4% had practiced self-help techniques. The participants with cancer at present were more likely to have visited a T&CM provider than participants with cancer previously (13.6% vs. 8.7%, *p* = 0.020). Among the participants with cancer at present, 6.4% reported to have consulted a TM provider, 5.8% had consulted an acupuncturist, while 4.7% had consulted other CM providers. Women were significantly more likely than men to have used acupuncture and self-help techniques. No significant gender differences were found regarding visits to other CM providers, TM providers nor use of herbal medicine/natural remedies.

**Conclusion:**

The findings are in line with previous research suggesting that both men and women use TM complementary to other CM modalities outside the official health care system. As herbal medicine might interact with conventional cancer treatment, health care providers need to discuss such use with their patients.

## Background

In Norway, approximately 30,000 people are diagnosed with cancer each year, more men (17,763) than women (15,064). Prostate (5118), breast (3402), lung (3080), and colon cancer (3003) are the most frequent cancer forms. Median age at diagnosis (all cancer sites included) is 69 years for both men and women. By the end of 2016 did 262,884 people in Norway live with cancer [1].

Traditional and complementary medicine (T&CM) is understood as medicine which is not covered by conventional medicine [2]. T&CM merges the terms traditional medicine (TM) and complementary medicine (CM). TM draws on a long history and is understood as “the knowledge, skills, and practices based on the theories, beliefs, and experiences indigenous to different cultures […], used in the maintenance of health as well as in the prevention, diagnosis, improvement, or treatment of physical and mental illness” [3]. The term “complementary medicine” refers to a broad set of health care practices that are not part of that country’s own tradition nor conventional medicine and are not fully integrated into the dominant health-care system [3].

The use of T&CM among cancer patients has increased worldwide during the last decades [4]. A systematic review and meta-analyses published in 2012 revealed that 40% of cancer patients used T&CM (*n* = 65,000) [4] with an estimate of 25% use in the 1970s and 1980s to more than 32% in the 1990s and to 49% after 2000. Highest use was found in North America (46%, studies published between 1984 and 2008) followed by Australia/New Zeeland (40%, 1986–2008) and Europe (34%, 1981–2008). A more recent study published in 2018, estimated that 30% of European cancer patients had used T&CM during the last 12 months [5]. The most commonly used T&CM was intake of substances thought to have healing potential (homeopathy, herbal medicine etc.) [5]. This is in line with research published in Norway in 2013 where 24.6% of the cancer patients reported to have used herbal medicine/natural remedies while 12.5% had visited a T&CM provider. Overall T&CM use within the last 12 months was reported by 33.8% of the participants with cancer [6].

Young to middle aged and highly educated female cancer patients are the most frequent users of T&CM [6–11]. Frequent use is also reported among patients with cancer related symptoms, metastatic disease, patients receiving only palliative treatment, and patients diagnosed with cancer more than 3 months previously [12]. The most common reasons for cancer patients’ use of T&CM are to increase the body’s ability to fight the cancer, to improve physical and emotional well-being, to provide hope, and to counteract negative effects from the tumour and medical treatments [13]. Best-experienced benefit from T&CM was to improve physical and emotional well-being [13]. Most cancer patients use T&CM in conjunction with conventional cancer treatment [14].

In Northern Norway, spiritual forms of T&CM are the most commonly used T&CM modalities, including the local form of traditional healing called “reading” where the healer read a prayer over the illness [15, 16]. This “reading” is used alone or together with elements from the nature such as rocks or water, or other remedies like steel or wool. When steel is applied, a knife is often used [17, 18]. Cupping therapy is also a part of the TM in Northern Norway [19] as well as use of medical plants [20, 21] and tare [18]. One of the specialties of the traditional healers in Northern Norway is to stop bleedings. This is used when people injure themselves or when they are in hospitals suffering from bleedings after childbirth or operations [17, 18]. The “reading” can be received as distant healing or by visiting a traditional healer who is mostly non-professional and a non-commercial. The ability to heal is normally inherited from an older family member who chose their successor among their younger relatives [18, 22, 23]. Health care providers in Northern Norway are generally positive and open minded to their patient’s use of TM. They consider it a tool that can help the patients to cope with severe illness [24]. TM is widely used in Northern Norway across all ethnicities, but more used among Sami (the indigenous population of Northern Norway) and Kvens (descendants of Finnish-speaking settlers) than Norwegians living in the same areas [25]. Associations for use of TM differ from use of CM. TM users tend to be older, have more severe health complaints, have lower education, and lower socioeconomic status compared to the users of CM [26].

In the cross-field between TM and CM are spiritual healing and Sami neoshamanism where the practitioners use elements from traditional Sami healing and pre-Christian practice of Sami shamanism, but in in contrast to TM providers, advertise and charge money for their services [27, 28]. Many TM providers show disrespect for these providers as they charge money for their services and share their knowledge to whoever is willing to pay. Most TM providers believe that God, as a gift of grace, gave them the ability to heal and that they can loose their ability to heal if they charge money for their services [17, 18, 29].

Many cancer patients do not communicate their use of T&CM to their conventional health care providers and few oncologists ask their patients about such use, leading to a risk of interaction between T&CM use and conventional cancer treatment [30].

In a national survey among 606 different health care providers in Norway, 94% of the medical doctors, 93% of the nurses, and 70% of the complementary therapists believed that complementary modalities could cause adverse effects, and that it was risky to combine complementary and conventional cancer treatments. The majority of the medical doctors (61%) and nurses (55%) would neither discouraged nor encouraged the use of complementary modalities if patients asked them for advice. Less than 1% of the complementary therapists would have discouraged the use of conventional cancer treatments [31, 32].

The aim of this study was to investigate the prevalence and associations for use of T&CM among cancer patients in the municipality of Tromsø, and to investigate the differences in T&CM use between people living currently with cancer, people with cancer previously but not now, and people without a history of cancer.

## Method

### Data

The Tromsø study is a longitudinal, cross sectional cohort study of the Tromsø population. Tromsø is the largest town in Northern Norway as well as a municipality. At the time of the study, 73,480 people lived in Tromsø [33], and 64,500 of these lived in the city centre. The population is increasing, partly due to a growing number of people moving from rural areas into the town [34]. The citizens are multi-ethnic. Most are Norwegians, but Tromsø has also traditional Sami settlements and a Sami and Kven population migrated from other areas in Northern Norway. Other ethnic groups also inhabit the municipality, mainly due to studies or employment at the university hospital and the university [33, 35–37]. The Tromsø population is younger and have a higher education compared to the Norwegian average, but is similar concerning employment rates and income [38].

### Participants

**Fig. 1:**
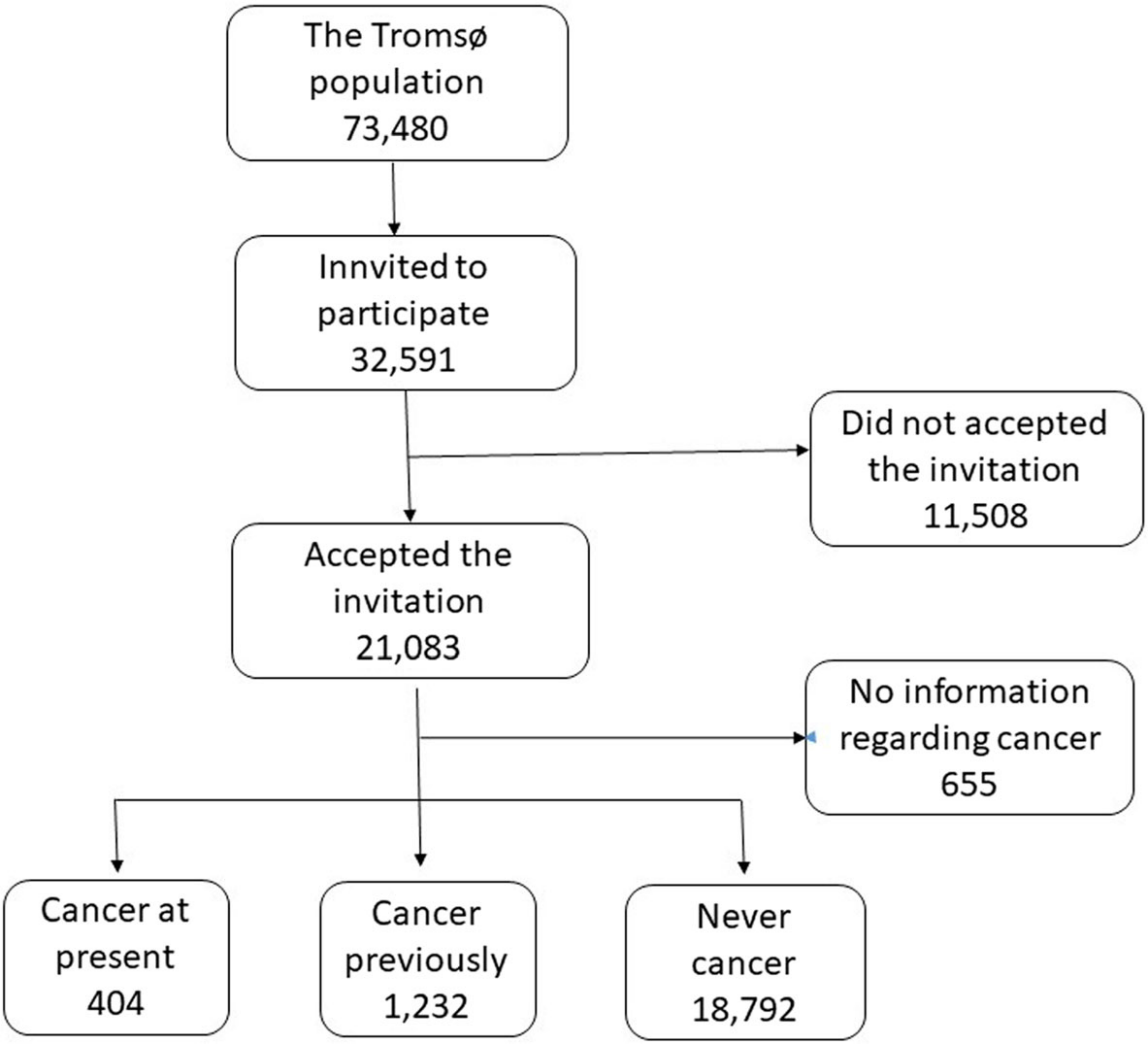
Flow chart of the study population

This study is based on questionnaire data from the 7th survey of the Tromsø Study conducted in 2015–2016. All inhabitants aged 40 and above were invited to participate (*n* = 32,591). All together 21,083 accepted the invitation, giving a response rate of 65%. By the time of the survey, 404 had cancer at present, 1232 reported to have had cancer previously but not now, while 18,792 had no history of cancer. A total number of 655 participants were excluded due to missing information about cancer (Fig. 1).

The Tromsø Study collected data through questionnaires, clinical examinations, and laboratory tests. The participants were recruited by a postal letter to all inhabitants aged 40 and above of the municipality of Tromsø. A comprehensive information brochure, as well as a four-page paper questionnaire (Q1) followed the invitation letter. Username and password to a digital version of the questionnaire did also follow. The participants could return the questionnaire by post or online. When the participants logged in, they found a questionnaire catalogue with two additional digital questionnaires; a second comprehensive questionnaire (Q2) and a body chart with questions about physical health such as pain, tiredness and exhaustion. At the clinical examination, the participants received a third digital questionnaire with questions about their diet (Q3). This survey was mostly answered on site of the clinical examination. If the participants needed assistance to complete the digital questionnaires, this was provided upon request.

### Variables

The data used in this study are collected in Q1 and Q2.

#### Health

Self-reported health was measured by two questions, one categorical in Q1 and a scale in Q2.

The first question: “How do you in general consider your own health to be?” had response categories “very bad”, “bad”, “neither good nor bad”, “good” and “excellent”, re-organized into “bad” (very bad and bad), “neither good nor bad” and “good” (good and excellent) (Q1). The request “We would like to know how good or bad your health is today” was measured by a scale numbered from 0 to 100 with 100 representing best possible health (Q2).

The question collecting data on cancer “Have you ever had, or do you have cancer?” offered the reply options “no”, “yes now” and “yes previously, but not now”. (Q1).

#### Traditional and Complementary Medicine (T&CM)

Use of T&CM providers was collected by a “yes” or “no” response to the Q1 questions: “Have you during the past year visited a traditional healer (helper, “reader” etc.)”, “Have you during the past year visited an acupuncturist?” and “Have you during the past year visited a CM provider (homeopath, reflexologist, spiritual healer etc.)” where of “Have you during the past year visited a traditional healer (helper, “reader” etc.)” was considered TM while “Have you during the past year visited an acupuncturist?” and “Have you during the past year visited a CM provider (homeopath, reflexologist, spiritual healer etc.)” were considered CM. Only modalities rooted in the Norwegian culture was considered TM in this study. Modalities considered TM in their home country other than Norway (like Traditional Chinese Medicine) was considered CM.

These questions regarding use of T&CM were organized together with questions asking for other health services (emergency room, general practitioner (GP), medical specialist, dentist, pharmacist, psychologist, psychiatrist, physiotherapist and chiropractor) in order to differentiate between T&CM provides and conventional providers. Chiropractors are considered conventional health care providers in Norway.

Use of herbal medicine/natural remedies and self-help techniques were collected through the Q2 questions “Have you used herbal medicines, natural remedies or herbal remedies during the last 12 months?” and “Have you used meditation, yoga, qi gong or Tai Chi as self-treatment during the last 12 months?” with the response options “yes” and “no”. No further description on how to understand herbal medicines, natural remedies and herbal remedies was provided, but use of cod-liver oil, Omega 3 fatty acids, vitamin D as well as a number of non-prescription and prescription drugs were asked for separately. Herbal medicine/natural remedies in Northern Norway can consist of plants and remedies that are used traditionally for medical purposes in Northern Norway (TM) like Angelica, Juniper, Stag’s-horn, Clubmoss, Pine, Rowan, Birch, Willow, Wolfsbane, Lingonberry, Lady’s mantle, Menyanthes, Peat moss, Iceland moss, Reindeer lichen, Fern, Spruce, Horsetail, Tormentil, Mezereum, Mountain sorrel, Sorrel, Alpine Blue Sow Thistle, Chaga mushroom, Hoof fungus, Marsh Labrador Tea, and tar [20, 21, 39–41], or remedies that are *not* part of the Northern Norwegian tradition (CM) like Ginger, Turmeric curcumin, Ginseng, Maidenhair tree*,* Green tea, Ashwagandha, and Reishi mushroom.

#### Other health services

The questions “Have you during the past year visited a general practitioner (GP)?”, and “Have you during the past year been admitted to a hospital?” were reported by the number of participants answering “yes” to the questions. The respondents answering “yes” to either of these questions were in addition asked to report the number of times they had seen the therapists during the last year.

#### Age, income, marital status, and education

Age per 31.12.2015 was measured continuously and reported as mean age with standard deviation (SD) as well as in the two categories “40–59 years” and “60 and above”.

Household income was measured by 7 response categories (“Less than NOK 150’/€ 15’” , “NOK 150’-250’/€ 15’-25’” , “NOK 251’-350’/€ 25.1’-35’” , “NOK 351’-450’/€ 35.1’-45’” , “NOK 451’-550’/ € 45.1’-55’” , “NOK 551’-750’/€ 55.1’-75’” ,“NOK 751′-1000′/€ 75.1′-100′” and “more than NOK 1,000’/€ 100’” ). These response categories were merged into the following three: “low income” (less than NOK 450′/€ 45′), “middle income” (NOK 450′-750′/€ 45′-75′) and “high income” (more than NOK 750′ /€ 75′). The question “How would you evaluate your finances?” had five response categories (“very good”, “good”, “average”, “difficult” and “very difficult”) which was collapsed into: “good” (very good and good), “average” and “difficult” (difficult and very difficult).

The questions “What is the highest level of education you have completed?” and “Do you live with a spouse/partner?” have all the response categories presented in Table 1.

**Table 1:** Basic characteristics of the participants

	The total population		Cancer at present		Cancer previously, not now		Never cancer		*p*-value
	%	(*n* = 20,428^c^)	%	(*n* = 404)	%	(*n* = 1232)	%	(*n* = 18,792)	
Age									
Mean (SD)	57.18 (11.365)		68.14 (10.945)		64.55 (10.945)		56.46 (11.117)		< 0.001^b^
40–59 years	61.2	12,179	20.9	83	33.2	399	63.9	11,697	< 0.001^a^
60 years and above	38.8	7711	79.1	315	66.8	802	36.1	6595	
Gender									< 0.001^a^
Women	52.3	10,674	43.8	177	56.1	681	52.2	9806	
Men	47.7	9754	56.2	227	43.9	541	47.8	8986	
Living with a spouse/partner									< 0.001^a^
Yes	77.0	14,907	71.4	270	72.7	856	77.4	13,781	
No	23.0	4448	28.6	108	27.3	322	22.6	4018	
Household income									
Low	22.1	4330	35.9	133	31.8	375	21.1	3822	< 0.001^a^
Middle	29.2	5742	34.3	127	31.2	368	29.0	5247	
High	48.7	9564	29.7	110	36.9	435	49.9	9019	
How will you evaluate your finaces									0.021^a^
Good	70.5	14,168	68.9	266	69.4	834	70.6	13,068	
Average	26.0	5223	28.5	110	28.4	341	25.8	4772	
Difficult	3.5	696	2.6	10	2.2	26	3.6	660	
Years of Education									< 0.001^a^
Primary school	22.7	4577	37.4	145	28.0	337	22.1	4095	
Secondary school	27.8	5588	25.8	100	24.2	291	28.0	5197	
College/university less than 4 years	19.5	3929	16.5	64	20.0	241	19.6	3624	
College/university 4 years or more	30.0	6026	20.4	79	27.8	335	30.3	5612	
Smoke daily									< 0.001^a^
Yes, now	13.8	2808	10.9	43	10.9	134	14.1	2631	
Yes, previously	44.1	8947	53.7	211	51.1	627	43.5	8109	
Never	42.0	8521	35.4	139	37.9	465	42.4	7917	
Frequency of drinking alcohol									< 0.001^a^
Monthly or less frequently	32.3	6569	43.3	173	34.7	427	31.9	5969	
More than once a month	67.7	13,777	56.8	227	65.3	802	68.1	12,748	
Frequency of exercise									0.555^a^
Less than weekly	15.9	3182	17.8	69	15.9	192	15.8	2921	
Weekly or more frequently	84.1	16,893	82.2	318	84.1	1012	84.2	15,563	
Ethnicity									0.096^a^
Norwegian	90.4	18,462	93.5	361	94.3	1133	92.3	16,968	
Sami/Kven	4.0	827	4.1	16	3.2	38	4.2	773	
Other	3.4	689	2.3	9	2.6	31	3.5	649	

^a^ Pearson’s chi-square test between the groups cancer at present, cancer previously not now, and never cancer; ^b^ One-way ANOVA test; ^c^ Due to missing responses to some of the questions, the number of respondents in single questions does not always add up to the total n

#### Lifestyle

To measure consumption of alcohol the question: “How often do you usually drink alcohol?” was used to separate the participants with a minimum of alcohol consumption from the participants drinking alcohol on a regular basis. The response category “never”, and “monthly or less frequently” were merged into “monthly or less frequently”, while the categories “2-4 times a month”, “2-3 times a week”, and “4 or more times a week” were merged into “more than once a month”.

Exercise was recorded through the question: How often do you exercise (i.e walking, skiing, swimming or training/sports)? with the response categories: “never”, “less than once a week”, “2-3 times a week” and “approximately every day”. These categories were merged in to “less than weekly” and “weekly or more frequently”.

The question “Do you, or did you smoke daily? have all the response categories presented in Table 1.

### Analyses

We used Pearson chi-square tests, Fisher exact tests, and one-way ANOVA tests to describe the basic characteristics of the participants and to calculate differences between the participants with cancer at present, the participants who have had cancer previously but not now, and the participants without a history of cancer (Table 1). For adjusted values (presented in the text only) we used binary logistic regressions. All analyses were conducted using SPSS for Windows (version 24.0, SPSS, Inc., Chicago, IL). The significance level was set to *p* < 0.05.

## Results

### Basic characteristics of the participants

The participants were all 40 years of age and above. Mean age was 57 years, with a significant higher age among the participants with cancer at present and the participants with previous cancer (68 and 65 years, respectively) compared to the participants with no history of cancer (56 years) (*p* < 0.001, Table 1). There were slightly more women participating than men (52% vs. 48%, *p* < 0.001); but more men than women with cancer (56% vs 44%, *p* < 0.001). Most of the participants lived with a spouse/partner (77%), but slightly fewer participants with cancer at present (71%) and with cancer previously (73%, < 0.001). Half of the participants (49%) had a high household income; however, this was not true for the participants with cancer at present (30%) and the participants with cancer previously (37%, < 0.001). Although there were differences in household income, their financial situation was similar for the majority of the participants (69%–71% of the participants, both with or without cancer, found their financial situation to be good, *p* = 0.021). While 50% of the participants had university education, this was only the case for 37% of the participants with cancer at present and 48% of the participants with cancer previously (*p* < 0.001). The participants with cancer at present and the participants with cancer previously were less likely to smoke daily than the group without cancer, but more likely to have smoked previously. The participants who never had cancer were most likely to be never smokers (*p* < 0.001). Those with cancer at present were, on the other hand, less likely to drink alcohol compared to participants with cancer previously and the group with no history of cancer. No associations were found regarding how often the participants exercised (*p* = 0.555). Most of the participants (84%) exercised at least once a week.

Most of the participants reported good health (69%) with a mean score of 76.24 on a 0–100 point scale where 100 was best possible health. This was mostly true for the participants with no history of cancer and cancer previously but not at present. Participants with cancer at present had significantly poorer health (only 46% with good health and a mean score of 65.7, *p* < 0.001, Table 2).

**Table 2:** Self-reported health and use of health care services among the participants

	The total population		Cancer at present		Cancer previously, not now		Never cancer		*p*-value
	% (*n* = 20,428^c^)		% (*n* = 404)		% (*n* = 1232)		% (*n* = 18,792)		
Self-reported Health (scale 0–100)									
Mean (SD)	76.24 (16.192)		65.7 (18.948)		73.09 (16.695)		76.66 (16.002)		< 0.001^b^
Self-reported health									< 0.001^a^
Good	68.9	13,971	45.5	181	60.7	741	69.9	13,049	
Neither	25.7	5215	41.7	166	32.6	398	24.9	4651	
Bad	5.4	1103	12.8	51	6.6	81	5.2	971	
T&CM provider	10.3	2052	13.6	51	8.7	102	10.3	1899	0.020^a^’
Acupuncturist	4.8	958	5.8	22	3.9	46	4.8	890	0.232^a^
Traditional healer	2.5	508	6.4	24	3.0	35	2.4	449	< 0.001^a^’
Other CM provider	5	1007	4.7	18	3.6	43	5.1	946	0.075^a^
Herbal medicine/natural remedies	17.0	3404	17.9	69	17.9	215	16.9	3120	0.625^a^
Self-help techniques	10.2	2053	6.4	25	9.3	111	10.4	1917	0.022^a^’
Over all use of T&CM	30.1	5926	33.4	123	30.3	352	30.0	5451	0.361^a^
Visit a GP last year	80.2	16,306	92.4	366	89.5	1096	79.3	14,844	< 0.001^a^
Number of visits to GP Mean (SD)	3.43 (3.556)		4.94 (4.848)		3.81 (3.306)		3.37 (3.530)		< 0.001^a^
Been hospitalized	10.8	2197	40.8	161	19.7	241	9.6	1795	< 0.001^a^
Visited an out-patient clinic	28.7	5728	66.1	248	49.2	587	26.6	4893	< 0.001^a^

^a^ Pearson’s chi-square test between the groups cancer at present, cancer previously not now, and never; ^b^ One-way ANOVA test; ‘The significant differences did not remain when adjusted for age and gender; ^c^ Due to missing responses to some of the questions, the number of respondents in single questions does not always add up to the total n

### Prevalence of T&CM use

Around one third (30.1%) of the participants had used T&CM, either consulted a T&CM provider (10.3%), used herbal medicine/natural remedies (17%), or used self-help techniques like meditation, yoga, chi gong or Tai Chi (10.2%). Participants with cancer at present were more likely to have consulted a T&CM provider than the participants without cancer (13.6% vs 10.3%). The participants with cancer previously were on the other hand less likely to have consulted a T&CM provider than the participants without cancer (8.7% vs 10.3%, *p* = 0.020, Table 2).

The participants in the study visited an acupuncturist on average 5.65 times, a traditional healer 2.48 times and other T&CM providers 4.47 times. There were no significant differences regarding number of sessions nor number of modalities used between participants with cancer at present, participants with cancer previously and participants with no history of cancer.

Participants with cancer at present were most likely to have seen a traditional healer (6.4%). This was also the only T&CM provider used more frequently by the participants with cancer at present, compared to participants with cancer previously and participants without cancer (6.4% vs 3% and 2.4%, *p* < 0.001). Acupuncture was used by 5.8% of the participants with cancer at present, 3.9% of the participants with cancer previously and 4.8% of the participants without cancer (*p* = 0.232). Use of other T&CM providers were reported by 4.7% of the participants with cancer at present, 3.6% of the participants with cancer previously and 5.1% of the participants without cancer (*p* = 0.075). No differences were found concerning use of herbal medicine/natural remedies where both participants with and without a history of cancer reported such use to some degree (17–18%, *p* = 0.625, Table 2). Similar use of herbal medicine/natural remedies was also found in men and women with cancer (18.3% vs 17.5%, *p* = 0.840). Women with cancer previously were, however, more likely to use herbal medicine/natural remedies than men were (19.8% vs 15.4%, *p* = 0.044).

Participants with cancer at present and participants with cancer previously were less likely to use self-help techniques than the population without cancer (6.4% and 9.3% vs 10.4%, *p* = 0.022, Table 2).

### Associations for T&CM use among participants with cancer at present

Women and participants with a Sami/Kven ethnicity were more likely to use T&CM than men and participants with other ethnicities (40%, *p* = 0.018 and 73.3%, *p* = 0.004 respectively, Table 3). No differences were found between users and non-users of T&CM regarding age, household income, education, self-reported health, frequency of alcohol consumption, daily smoking nor exercise in participants with cancer at present (Table 3).

**Table 3:** Associations for T&CM use among participants with cancer at present

	No T&CM		Any T&CM		*p*-value
Age					
Mean age (SD)	68.37 (9.772)		66.85 (11.487)		0.189^c^
	**%**	**n**	**%**	**n**	
40-59 years	58.4	45	41.6	32	0.105^a^
60 years and above	68.4	195	31.6	90	
Gender					0.018^a^
Women	59.7	92	40.3	62	
Men	71.5	153	28.6	61	
Ethnicity					
Norwegian	68.5	233	31.5	107	0.004^b^
Sami/Kven	26.7	4	73.3	11	
Other	75.0	6	25.0	2	
Household income					0.861^a^
< NOK 450’/ € 45’	66.4	79	33.6	40	
NOK 450’-750’/€ 45’-75’	66.7	78	33.3	39	
>NOK 750’/€ 75’	69.5	73	30.5	32	
Years of education					0.747^a^
Primary school	63.8	83	36.2	47	
Secondary school	66.7	60	33.3	30	
College/university less than 4 years	68.3	41	31.7	19	
College/university 4 years or more	71.2	52	28.8	21	
Self-reported health					0.788^a^
Good	67.9	114	32.1	54	
Neither	64.7	97	35.3	53	
Bad	68.9	31	31.1	14	
Smoke daily					0.878^a^
Yes, now	69.0	29	31.0	13	
Yes, previously	65.8	127	34.2	66	
Never	68.0	85	32.0	40	
Frequency alcohol consumption					0.350^a^
Monthly or less frequently	64.0	96	36.0	54	
More than once a month	68.7	147	31.3	67	
Frequency of exercise					0.215^a^
Less than weekly	73.8	48	26.2	17	
Weekly or more frequently	65.9	197	34.1	102	

^a^ Pearson chi square test; ^b^ Fisher exact test; ^c^ ANOVA test; Any T&CM use are use of either T&CM provider, herbal medicine/natural remedies or T&CM self-help techniques.

We found only small differences between men and women with cancer at present regarding use of T&CM *providers*. The only significant gender difference found was regarding use of acupuncture where 9.5% of the women reported such use compared to 3.2% of the men (*p* = 0.010). No significant differences were found between men and women with cancer at present regarding use of traditional healing (7% vs 5.9%, *p* = 0.675) and other complementary modalities (5% vs 4.5%, *p* = 0.837). This was also the case for herbal medicine/natural remedies were 18.3% of the men and 17.9% of the women reported such use (*p* = 0.840). Use of T&CM self-help techniques was more frequently used by women with cancer at present (13.2%) compared to men (1.4%, *p* < 0.001).

## Discussion

### Main findings

This study revealed that one third of the participants with cancer at present had used some kind of T&CM. Most frequently used were herbal medicine and natural remedies, followed by traditional healing, and self-help techniques. Women used acupuncture and self-help techniques more often than men, and were therefore more frequently users of T&CM in general.

We found no differences in overall use of T&CM between participants with cancer at present, cancer previously and participants without cancer. Visits to a T&CM provider on the other hand, were more frequent among participants with cancer at present, particularly visits to traditional healers. Self-help techniques were most frequently used by participants without cancer at present.

Participants with cancer at present differed significantly from participants without cancer and cancer previously by being older, male, having lower household income, lower education, and poorer self-reported health. They were more likely to have smoked previously and to drink alcohol monthly or less frequently.

### Overall T&CM use including use of a T&CM provider, herbal medicine/natural remedies and self-help techniques

The findings of no significant differences between participants with cancer at present or previously, and participants with no history of cancer regarding overall use of T&CM, are in line with findings from the 6th survey of the Tromsø study conducted in 2008 [6, 42], but in contrast to other studies indicating that cancer patients use more T&CM than people without cancer [43–45]. One reason for the lack of differences in the present study might be that the participants with cancer at present seem more prone to use T&CM providers, but less likely to participate in self-help techniques like meditation, yoga, tai chi and qi gong. Another reason might be that T&CM is used also for less severe illnesses than cancer and for prevention of disease and well-being [46, 47]. Also, the fact that there were more men in the cancer group might have influenced as men are known to use T&CM less frequently that women [6].

The overall use of T&CM among patients with cancer at present (33.4%) was somewhat higher than what was found among Swedish cancer patients (26%, published in 2019) [48] but lower than what was found in Denmark (49.4%, published in 2014) [14], North America (46%, published 2012) and Australia/New Zeeland (40%, published 2012) [4]. It was similar to Scandinavia (36%, published 2016) [49] and Europe as a whole (30%, published 2018) [5], and similar to the 6th survey of the Tromsø study conducted in 2007/2008 [6]. The wide range in reported use of T&CM among cancer patients worldwide could be due to different traditions for T&CM use, different policy of implementing T&CM in conventional cancer care, different availability of conventional health care, differences in the definition of TM, CM and CAM, and/or differences in time when the studies were conducted [50].

### Use of T&CM providers

The finding of higher use of T&CM providers among participants with cancer at present than the participants who never have had cancer, is not in accordance with findings from the 5th survey of the Tromsø study conducted in 2002, where no differences were found regarding use of T&CM providers between participants with and without cancer [51]. One reason for this might be that participants with previous and present cancer were combined in the same category in the 5th survey of the Tromsø study. This is suspected, as participants with cancer previously in the present study were less likely to have seen a T&CM provider than participants with cancer at present as well as participants without a history of cancer. If we had combined participants with cancer at present and cancer previously, there would have been similar use in the cancer group and the non-cancer group in this study as well.

The finding of 13.6% use of T&CM providers among the participants with cancer at present is on the other hand in accordance with use found among participants with present or previous cancer in the 6th survey of the Tromsø study [6]. As only 8.7% of participants with previous cancer reported use of a T&CM provider in the present study, this shows a decrease of such use since 2008. The reason for that is not clear, other than that use of T&CM in general has decreased in Norway in recent years [52].

The higher use of TM providers among the participants with cancer at present than among the participants with no cancer and cancer previously is in accordance with earlier findings showing that hospitalized patients in poor health use TM providers to a much larger degree than those not being hospitalized [17, 25]. Previous research show that TM providers are frequently called upon in Northern Norway when serious disease occur, used as an additional resource/coping strategy for the patients and their families, especially in Sami populations [17, 24, 53]. A previous study of Norwegian cancer patients also shows that cancer patients with a poor prognosis (less than 20% expected 5-year survival at time of diagnosis) visit T&CM provider to a higher degree than cancer patients with a better prognosis (40–60% expected 5-year survival) [54]. In late state cancer and palliative care, patients need strategies in coping with their life challenges and disease where TM is one way to manage. In the palliative stage, conventional health care providers are ethical obligated to do good and treat people holistically. It is important that they delve more deeply into the philosophical underpinning of the patients viewpoint and respect their choice of using T&CM [55, 56]. As traditional healing has strong culture traditions and is recognized in the local communities [57] this is frequently used when the health care system can no longer give comfort.

### Associations for overall use of T&CM (provider, herbal medicine/natural remedies or self-help techniques)

The findings of more over-all use of T&CM in women with cancer than men is in line with most national [6, 54] and international [9, 58–60] studies. The reason for this might be that women with cancer experience unmet health care needs within conventional health care [61, 62] and that men, who have a tendency to see the body as more mechanical [62], to a lager degree have their health care needs met within conventional health care [42]. Women are also more likely to undertake health care visits in general than men [63–65]. Like our study, previous studies found that women with cancer are more likely to report over all T&CM use. Once the T&CM modalities are split up, men and women equally initiate all therapies except for psychotherapy and mind-body approaches like yoga and meditation [66].

The findings of no association regarding age, education and household income and use of T&CM are in contrast to a systematic review investigating associations for cancer patients use of complementary and alternative medicine (CAM) [9]. A possible reason for this discrepancy might be that we included traditional medicine (TM) in our study, and that users of TM are known to have other associations for use than CM modalities not part of the country’s own tradition. As mentioned in the background section are users of TM older, have lower socioeconomic status and more severe health complaints than users of CM [26].

We did not find associations for health parameters like self-reported health, exercise, smoking habits, or alcohol intake and use of T&CM. This indicates that patients living with cancer do use T&CM regardless of other health approaches. This is not in accordance with previous findings suggesting that non-smoking cancer patients [67, 68] with poorer health [9, 54], who exercised more frequently [68] are more likely to have used CM. One reason for this discrepancy might be that the participants with cancer at present already were more likely than the other groups to have quit smoking and to drink alcohol less frequently, and that exercise along with reduced alcohol consumption and T&CM use are the most commonly stated changed behaviours after cancer diagnosis [69].

### Risk connected to use of T&CM

Eighteen percent of the cancer patients in this study reported to have used herbal medicine and natural remedies. Despite the fact that T&CM is considered natural and therefor associated with low risk [30], use of T&CM is associated with direct as well as indirect risk for cancer patients [70, 71]. Herbs like Turmeric, Green tea, Ginger, Ashwagandha and Reishi mushroom are examples of herbal medicine that can influence cancer and the conventional treatment of cancer [72]. The direct risk of negative interaction between herbal medicine and conventional cancer treatments increases when the patients do not discuss their use of T&CM with their oncologist.

### Implications of the findings

This is the first study in Norway to compare T&CM used by people with cancer at present to T&CM used by people who have had cancer previously. In two previous studies [6, 51] the use of T&CM were found to be similar in cancer patients and the population without cancer [42]. This has led us to believe that cancer patients in Norway have similar use of T&CM as the general population. When participants with cancer at present were analysed separately from participants with cancer previously, we found that participants with cancer at present were more likely to have seen a T&CM provider, and that participants with cancer previously were less likely to have seen a T&CM provider than those who never experienced cancer. This means that health care providers need to be extra aware of use of T&CM in patients who have cancer at present, particularly use of traditional and herbal medicine, as neither the patients nor the conventional health care providers seems to take initiative to discuss this topic [32]. This lack of communication can increase the risk of negative interaction between T&CM and conventional cancer care as herbal medicine, used by 18% of the participants with cancer at present, is known to interact with conventional cancer treatment. Another study separating users of traditional medicine from users of other complementary therapies [26], found that the users of traditional medicine differed significantly from the users of other complementary modalities by being older and have lower socio-economic status. We found in addition that men with cancer was just as likely to use TM as women were. Health care providers need therefor to have an extra focus of possible use of TM and herbs in patient groups who are not considered typical users of complementary therapies.

### Strengths and limitations of the study

The main strengths of this study is the high number of participants representing the whole target population rather than a random sample, and the rather high response rate of 65%. Despite this, the generalizability of the findings might have been affected as the non-responders differed from the responders regarding age and gender with higher response rate among women [6]. The fact that only 404 participants had cancer at present and only 123 had used T&CM made the material unsuitable for sub-group analyses regarding different T&CM modalities.

One of the limitations is the self-reported T&CM, leading to possible bias concerning how to understand T&CM and recall of use. We argue, however, that the examples of T&CM provided in the questionnaire would give the participants a rather clear idea of how to understand T&CM, partly because several other health care services were asked for in the same section. This is also the case for “herbal medicines, natural remedies and herbal remedies” where cod-liver oil, Omega 3 fatty acids, vitamin D as well as a number of non-prescription and prescription drugs were asked for separately. Vitamins and minerals in general were not asked for explicitly, consequently we cannot exclude the possibility that such use has been reported alongside use of for “herbal medicines, natural remedies and herbal remedies” leading to an increased proportion of such use reported in the study.

As the recall time was only 12 months, the recall bias is limited and further equally distributed among participants with cancer at present and participants with no cancer or cancer previously. Recall bias might also have influenced the self-reported cancer as a previous study shows that self-reported cancer might differ from cancer registered in the Cancer Registry of Norway [51]. We believe that this is most true for the participants with cancer previously and not for the participants with cancer at present. We can therefore not exclude the possibility that participants with cancer previously might occur in the never cancer group.

## Conclusions

One third of the participants with cancer at present reported to have used T&CM within the last year, in particular traditional healing and herbal medicine/natural remedies. Participants with cancer at present were more likely to have seen a T&CM provider than the participants without cancer and with cancer previously. The cancer patients seem to employ parallel health care, including conventional as well as traditional and complementary medicine. Both men and women were frequent users of traditional healing and herbal medicine/natural remedies. As herbal medicine might interact with conventional cancer treatment, health care providers need to discuss such use with their patients and be aware of the fact that traditional healing and herbs are used by patients not earlier known as typical T&CM users.

## Data Availability

The raw dataset is not available due to Norwegian privacy regulations. Applicants for any data must be prepared to conform to Norwegian privacy regulations.

## Abbreviations

€: Euro
CAM: Complementary and Alternative Medicine
CM: Complementary Medicine
NOK: Norwegian Kroner
REK: Regional Committee of Medical and Health Research Ethics
SD: Standard Deviation
T&CM: Traditional and Complementary Medicine
TM: Traditional Medicine
UiT: University of Tromsø
